# Retraction Note: Efficient administration of a combination of Nifedipine and sildenafil citrate versus only Nifedipine on clinical outcomes in women with threatened preterm labor: a systematic review and meta-analysis

**DOI:** 10.1186/s12887-026-06556-5

**Published:** 2026-02-07

**Authors:** Elham Manouchehri, Somayeh Makvandi, Mahdieh Razi, Maryam Sahebari, Mona Larki

**Affiliations:** 1 Rheumatic Diseases Research Center, Mashhad University of Medical Sciences, Mashhad, Iran; 2 Department of Midwifery, Faculty of Nursing and Midwifery, Mashhad Medical Sciences, Islamic Azad University, Mashhad, Iran; 3 Menopause Andropause Research Center, Ahvaz Jundishapur University of Medical Sciences, Ahvaz, Iran; 4 Department of Pediatrics, School of Nursing and Midwifery, Mashhad University of Medical Sciences, Mashhad, Iran5Nursing and Midwifery Care Research Center, Mashhad University of Medical Sciences, Mashhad, Iran; 5 Nursing and Midwifery Care Research Center, Mashhad University of Medical Sciences, Mashhad, Iran; 6 Department of Midwifery, School of Nursing and Midwifery, Mashhad University of Medical Sciences, Mashhad, Iran

**Retraction Note: BMC Pediatrics (2024) 24:106**

**https://doi.org/10.1186/s12887-024-04588-3**

The Editor has retracted this article because one of the studies (Maher et al. 2019, reference 26 in the original article) that was included in this Systematic Review and meta-analysis has been retracted. The Editor therefore no longer has confidence in the results and conclusions of this article. The authors have been offered the opportunity to submit a revised manuscript, excluding the retracted study, to the journal.

Author Mona Larki did not explicitly state whether or not they agree with this retraction. Authors Elham Manouchehri, Somayeh Makvandi, Mahdieh Razi and Maryam Sahebari did not respond to correspondence from the Publisher about this retraction.

